# Supplementary material for: Genomic factors shaping codon usage across the Saccharomycotina subphylum
Source: G3 (Bethesda). 2024 Aug 30;14(11):jkae207. doi: 10.1093/g3journal/jkae207 (PMC11540330; doi:10.1093/g3journal/jkae207)
Supplement: jkae207_Supplementary_Data [file jkae207_supplementary_data.zip › Supplemental_Material_Legends_G3-2024-405191.docx]

**Supplemental Material Legends**

**Table S1: Table of yeast genomes and features**. This table includes the origin of the yeast genomes, updated species names, and the examined genomic features.

**Table S2: RSCU of all codons across yeasts.** For each of the 1,154 yeast genome annotations, we report the mean RSCU across all coding sequences for all 61 codons.

**Table S3: Variable importance of random forest algorithm**: Importance values extracted from the random forest algorithm, which classified yeasts into orders based on RSCU. The higher the importance value the more informative it was in building the random forest.

**Table S4: Results of feature versus RSCU comparisons**: The RSCU for each codon was compared to yeast features. The p-value slope, intercept, lambda value, R-squared, and adjusted R-squared are reported for each comparison.

**Table S5: Results of pairwise RSCU comparisons**: The RSCU for each codon was compared to every other codon using a PGLS. The p-value, slope, lambda, R-squared, and adjusted R-squared are reported for each comparison.

**Table S6: Results of pairwise comparisons between S-value and other features**: The S-value was compared to other features using a PGLS. This also includes the other feature by feature comparisons. The p-value and slope are reported for each comparison.

**Table S7: Additive PGLS models**. We conducted all possible iterations of additive models to explain S-value based on genomic and assembly metrics. The models' AIC and BIC values are reported and used to select the most informative model accounting for the additional parameters.

**Table S8: Analysis of mitochondrial tRNAs**: This table includes the output from tRNAscan-SE from the available mitochondrial genomes of the *Hanseniaspora*.

**Table S9: All tRNAs with a predicted CGN anticodon within the *Hanseniaspora*.** tRNAscan-SE predicts the majority of tRNAs containing a CGN anticodon to be non-functional. This can be seen in the isotype prediction.

**Table S10: Presence of *TAD*-encoded tRNA modification enzymes in the *Hanseniaspora* and relatives.** The total number of K15440 (TAD1), K15441 (TAD2), and K15442 (TAD3) coding sequences are reported for each species.

**Table S11: *Hanseniaspora* transcriptomics BLAST results.** The highest BLASTx and BLASTn hits from transcripts with many and with no CGN codons.

**Table S12: Conservation of CGN codons in the *Hanseniaspora.*** Number of CGN codons in conserved arginine positions reported for the *Hanseniaspora* and their relatives.

**Figure S1: Variation in Relative Synonymous Codon Usage (RSCU) across the yeast subphylum.** Codons in blue are non-preferred, while codons in red are preferred. The third codon position is colored by GC- (green) or AT- (purple) ending codons. The x-axis is clustered based on a hierarchical clustering of RSCU values. The y-axis is sorted by Saccharomycotina order. Generally, GC- and AT-ending codons are clustered together. The exceptions are the general preference for the TTG codon and avoidance of CTT, CGT, GTA, ATA, CTA, and CGA codons.

**Figure S2:** Random forest classifier successfully classifies the majority of the withheld strains using genome-wide RSCU values. A) The confusion matrix of the testing species. The largest number of misclassified yeasts were assigned to the Alloacoideales. B) The relative importance of the codons with the highest importance.

**Figure S3:** Correlation between RSCU of all codons and yeast features. Raw p-values are shown on the scale, with positive correlations shown in blue and negative correlations shown in red. More significant correlations are darker with larger circles. Significant raw p-values are shown with stars at the levels 0.05, 0.01, and 0.001.

**Figure S4:** Correlation between RSCU of all possible codons. Raw p-values are shown on the scale, with positive correlations shown in blue and negative correlations shown in red. More significant correlations are darker with larger circles. Generally, AT- and GC-ending codons are correlated within groups and anti-correlated across groups. The coding sequence with the most unexpected correlations is CTA (leucine).

**Figure S5:** Correlation between features of the 1,154 yeasts. Raw p-values are shown on the scale, with positive correlations shown in blue and negative correlations shown in red. More significant correlations are darker with larger circles. Significant raw p-values are shown with stars at the levels 0.05, 0.01, and 0.001. S-value has significant positive correlations with genomic tRNA pool, total BUSCO count, complete BUSCO count, single copy BUSCO count, and N50. S-value has significant negative correlations with fragmented BUSCO count, missing BUSCO count, total number of contigs, and number of coding sequences.
