# Supplementary figures and images for: Genomic factors shaping codon usage across the Saccharomycotina subphylum
Source: G3 (Bethesda). 2024 Aug 30;14(11):jkae207. doi: 10.1093/g3journal/jkae207 (PMC11540330; doi:10.1093/g3journal/jkae207)

Heatmap Average RSCU values

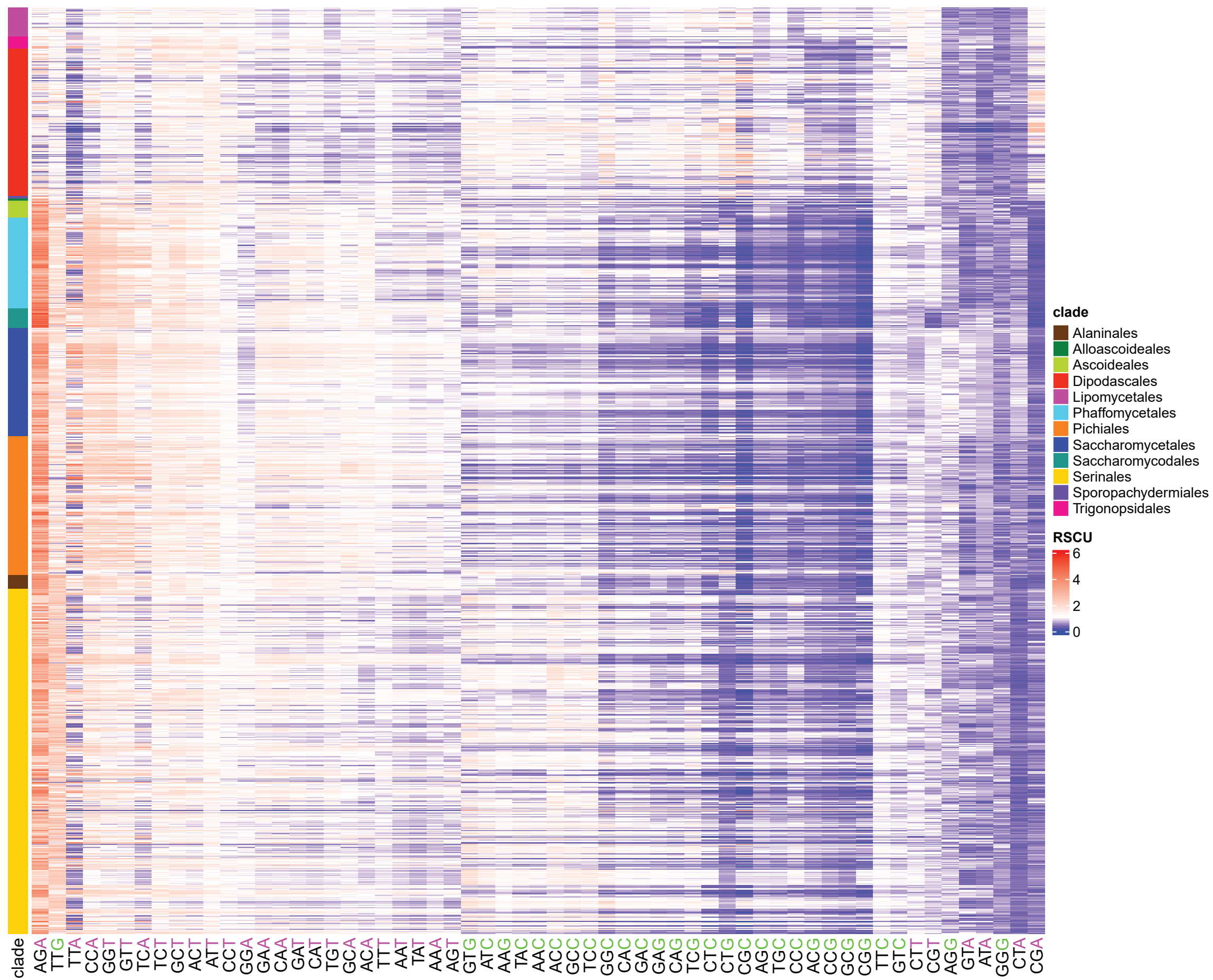

Supplement: jkae207_Supplementary_Data [file jkae207_supplementary_data.zip › Figure_S1_G3-2024-405191.pdf]

A

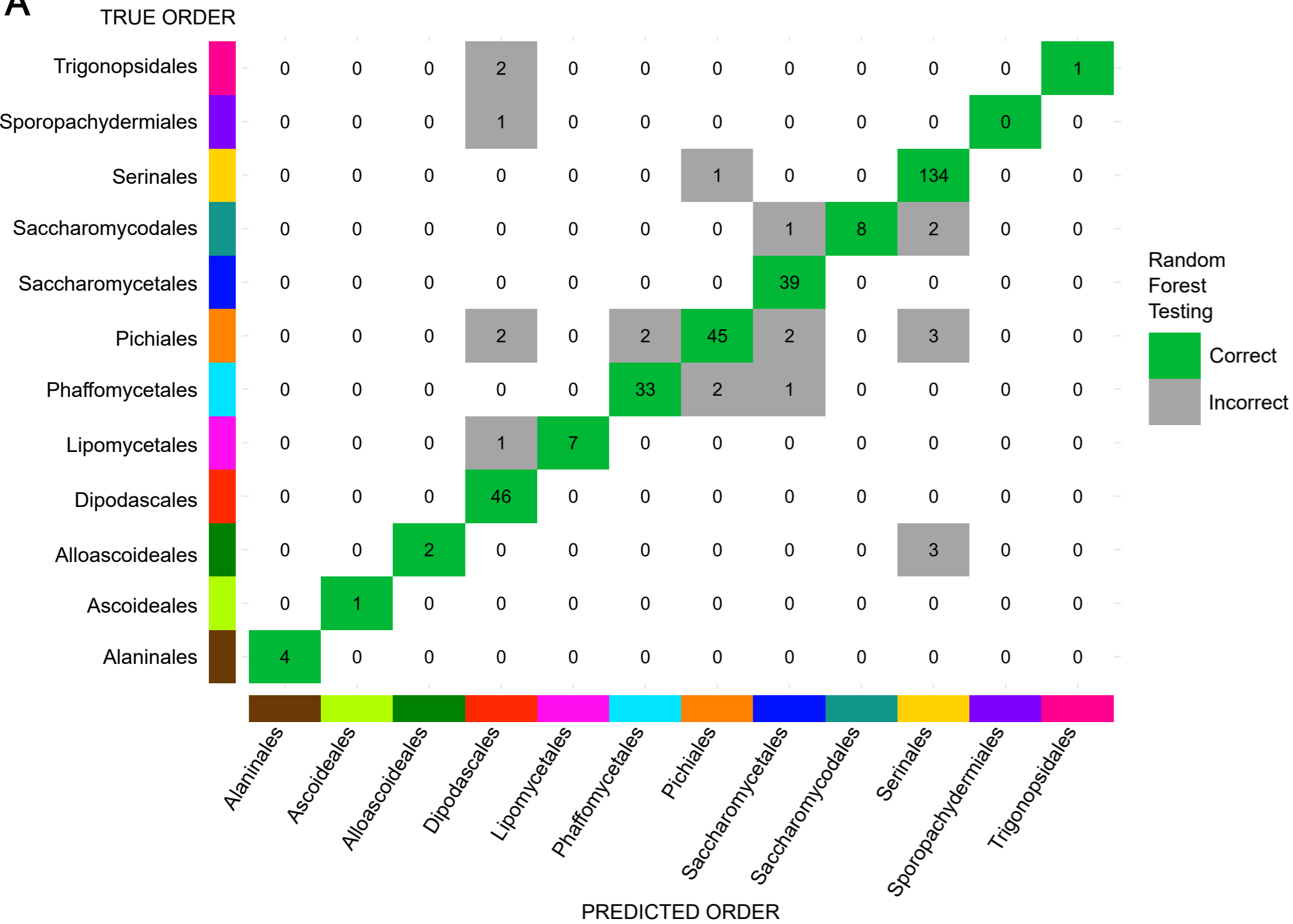

B

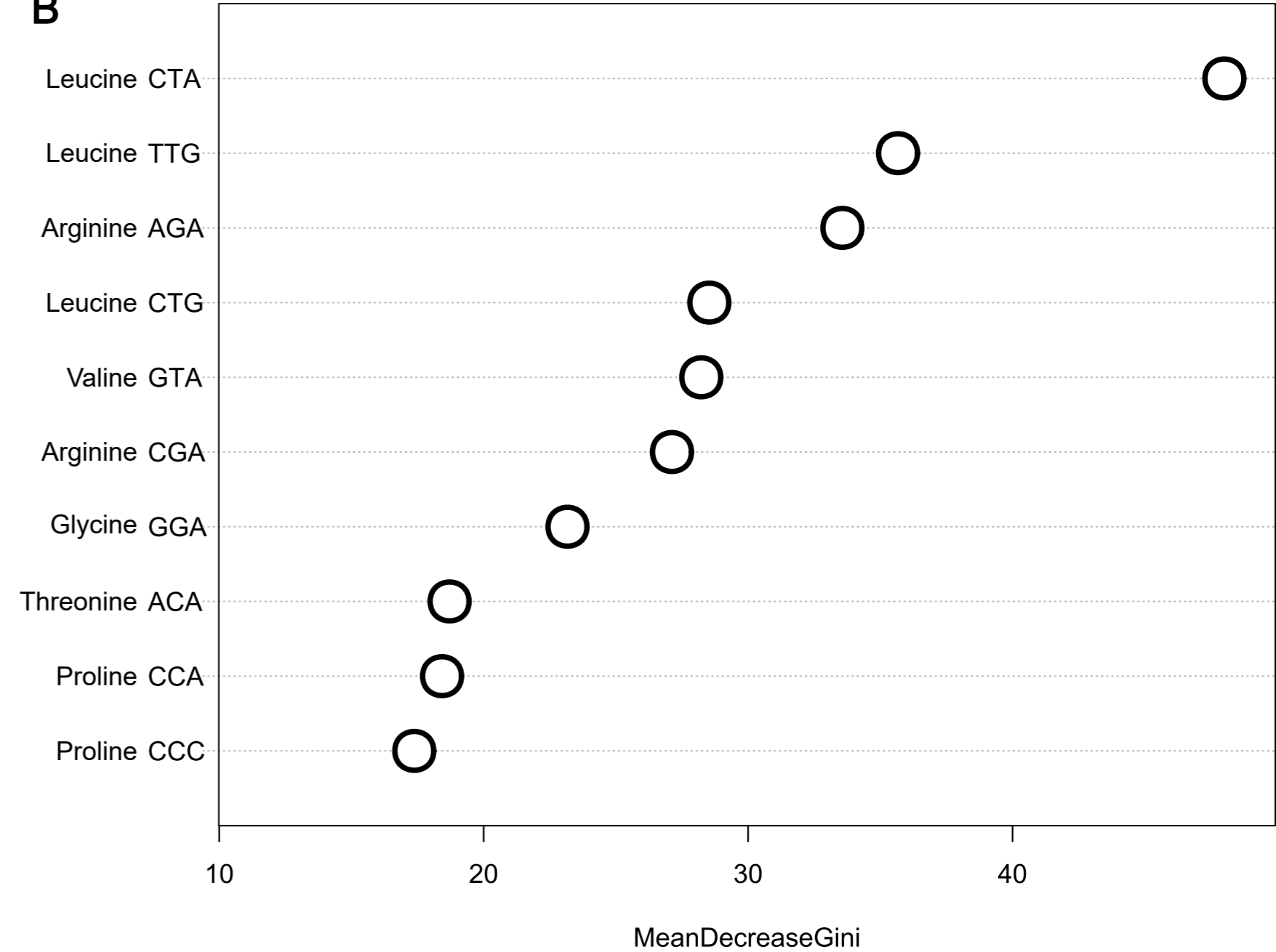

Supplement: jkae207_Supplementary_Data [file jkae207_supplementary_data.zip › Figure_S2_G3-2024-405191.pdf]

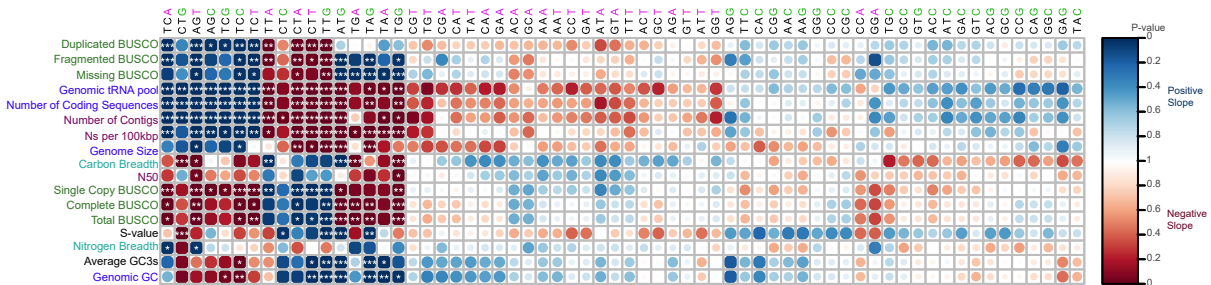

Supplement: jkae207_Supplementary_Data [file jkae207_supplementary_data.zip › Figure_S3_G3-2024-405191.pdf]

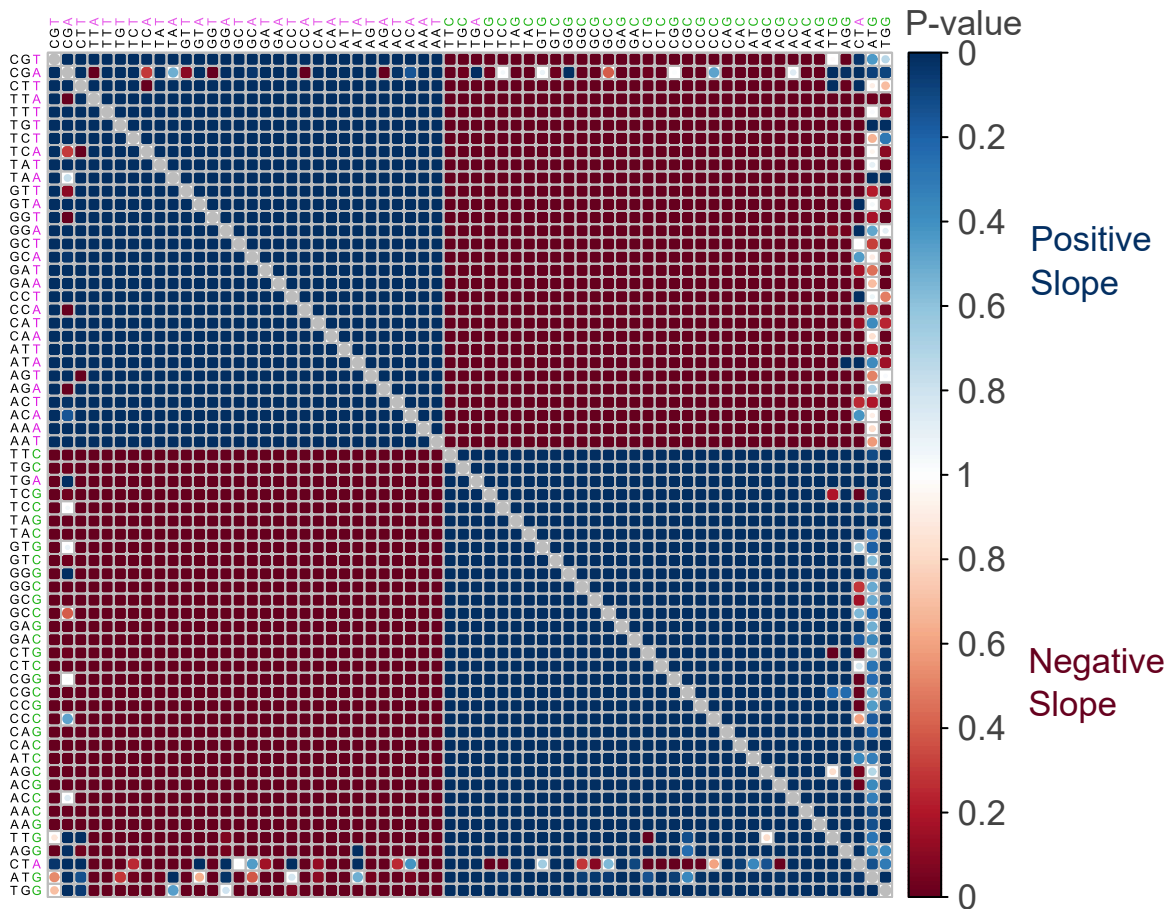

Supplement: jkae207_Supplementary_Data [file jkae207_supplementary_data.zip › Figure_S4_G3-2024-405191.pdf]

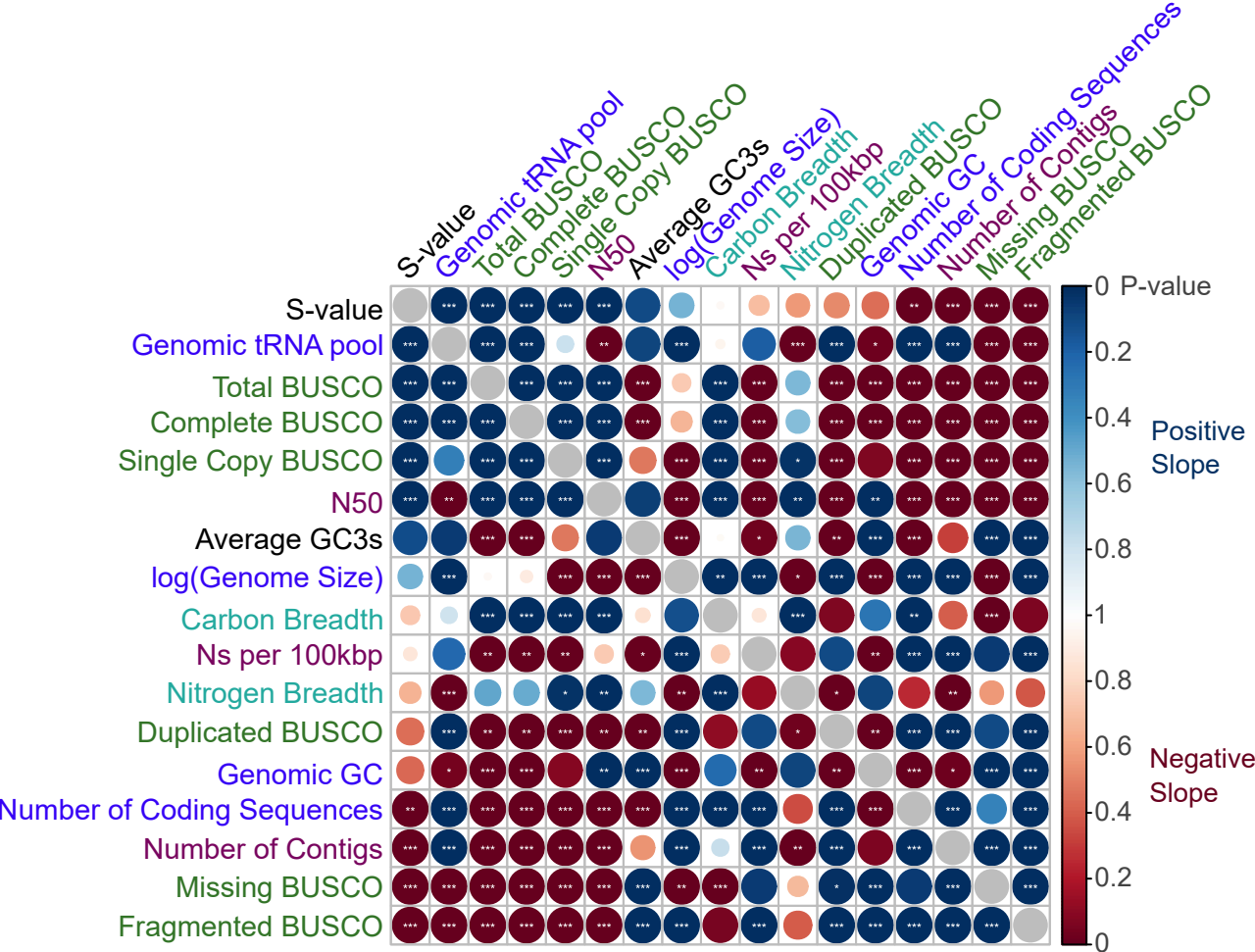

Supplement: jkae207_Supplementary_Data [file jkae207_supplementary_data.zip › Figure_S5_G3-2024-405191.pdf]
